# Axial Length of the Eyeball Is Important in Secondary Dislocation of the Intraocular Lens, Capsular Bag, and Capsular Tension Ring Complex

**DOI:** 10.1155/2016/6431438

**Published:** 2016-03-16

**Authors:** Anna Klysik, Katarzyna Kaszuba-Bartkowiak, Piotr Jurowski

**Affiliations:** Department of Ophthalmology, Medical University of Lodz, Zeromskiego 113, 90-549 Lodz, Poland

## Abstract

*Purpose*. To analyze the patients with secondary dislocation of CTR and IOL within 5 years from cataract surgery, to determine predisposing factors.* Methods*. 16 eyes of 15 patients aged 66.2 ± 6.7 (from 49 to 82) with CTR/IOL complex dislocation within 5 years from cataract surgery were compared with 26 patients aged 67.1 ± 7.2 (from 53 to 85), implanted with CTR during cataract surgery to manage zonule dehiscence and did not dislocate for at least 5 years, in respect of cause, axial length and IOL power, refraction, coexistent pathology, and trauma.* Results*. Axial length of the eyeball was 23.8 ± 1.3 (from 21 to 29) in the group of patients with CTR/IOL dislocation and 20.7 ± 1.2 (from 19 to 24) in patients with no dislocation present (*p* = 0.008). Crystalline lens dislocation was diagnosed before surgery in 13 of 16 patients with CTR/IOL complex dislocation as opposed to 7 of 26 eyes in the control group (*p* = 0.01). Pseudoexfoliation was present in 50% and 58% in both groups, respectively. Traumatic dislocation was present in 8 patients, none of them with CTR/IOL dislocation (*p* = 0.04).* Conclusion*. Longer axial length may contribute to the failure of the CTR to prevent in-the-bag IOL dislocation. Traumatic dislocation appears to be well fixed with the CTR.

## 1. Introduction

Dislocation of an intraocular lens (IOL) within the capsular bag is a rare, late complication of cataract surgery [1]. Capsular tension rings (CTR) probably reduce the frequency of in-the bag IOL dislocation but fail to prevent this complication fully [2]. Frequency of in-the-bag IOL dislocation increased with the development of cataract surgery technique [3].

Previously reported factors associated with CTR and IOL dislocation are pseudoexfoliation, uveitis, previous vitreoretinal surgery, high myopia [4], and trauma [2]. This factors are identical in secondary in-the bag lens dislocation, without CTR support.

It is not known which patients with predisposing factors are likely to benefit from CTR insertion and which patients require alternative IOL fixation.

The CTR device was introduced by Hara and Yamada [5], in the early 1990s, to stretch the lens capsule and retain the circular contour of the capsular bag equator after cataract removal, to prevent intraocular lens decentration and dislocation. Since then, various designs of CTRs have been in use [6]. They are indicated in eyes with zonular weakness or dehiscence, including those with pseudoexfoliation syndrome, high myopia, mature cataract, and subluxated lenses, from Marfan syndrome, Ehlers-Danlos syndrome, and other genetic diseases [7, 8]. Capsular tension rings reduce capsular bag shrinkage [9]. There are fewer higher-order aberrations in eyes with a multifocal IOL and a CTR than in eyes with a multifocal IOL only [10].

CTR and IOL complex dislocation requires further surgical approach. Management includes CTR removal, IOL exchange, replacement with an anterior or a sutured posterior chamber IOL, or suturing the IOL through the bag to the iris or the sclera [11, 12].

Pseudoexfoliation syndrome has been the most frequently indicated factor in secondary in-the-bag IOL dislocation [3].

Previously reported rate of CTR and IOL complex dislocation was 0.76% [2].

Retrospective series of 86 patients revealed that neither lens material nor lens design appears to play a role [13]. The mean time from surgery to spontaneous IOL dislocation was 8.5 years.

Retrospective series available in the literature are very difficult to compare, as CTR insertion rates vary greatly between surgeons and depend on individual surgeon's experience and approach to dislocated lens.

It would be a great advantage to be able to select the patients with zonule insufficiency, who can be fixed with CTR, from those, who require alternative IOL fixation.

We performed an analysis of the eyes with secondary IOL/CTR complex dislocation within 5 years from surgery. Control group consisted of the eyes, where CTR was inserted and did not dislocate over the period of 5 years.

## 2. Materials and Methods

Operating notes of 15 835 intraocular surgeries performed between January 2010 and December 2014 at the Department of Ophthalmology of the Medical University of Lodz were reviewed.

19 eyes of 17 patients' surgeries included CTR/IOL complex removal or refixation, including removals associated and not associated with secondary lens implantation.

16 eyes of 15 patients (10 males and 5 female) aged from 49 to 82 years (mean 66.2 ± 9.5) were analyzed. Three eyes met exclusion criteria. In 2 eyes the time from primary cataract surgery was more than 5 years and one patient suffered blunt eye trauma, before CTR/IOL complex dislocation was diagnosed.

To select control group, we reviewed the notes of 10 789 cataract surgeries performed at the Department of Ophthalmology of the Medical University of Lodz between January 2007 and August 2010. In 114 eyes of 106 patients, CRT were implanted. We identified 54 patients, remaining under the care of our out-patient department, who did not present with secondary IOL dislocation, for 5 or more years. Age and sex matched group of 26 eyes of 26 patients (17 male and 9 female) aged from 56 to 85 (mean 68.9 ± 7.2) were randomly selected from 54 and analyzed as a control group.

16 eyes of 15 patients, who presented with secondary CTR/IOL complex dislocation within 5 years from primary surgery, were analyzed in respect of

1. cause of crystalline lens dislocation;
2. preoperative or intraoperative diagnosis of lens dislocation or zonule dehiscence;
3. axial length and IOL power;
4. pre-op refractive status;
5. coexistent pathology.

Exclusion criteria were CTR/IOL dislocation more than 5 years from primary surgery and blunt eye trauma as a cause of CTR/IOL complex dislocation. We did exclude patients in whom trauma was the reason of crystalline lens subluxation before primary cataract surgery. The data were analyzed by means of descriptive statistics, and Fisher's exact test and chi-squared test were used for significance. In all analyses, a statistically significant difference was assumed for *p* ≤ 0.05.

## 3. Results

Axial lengths of the eyeball, refractive status, IOL power, and timing of the diagnosis of zonule dehiscence and subluxation in analyzed groups are summarized in Table 1.

In the group of patient with dislocation the axial length was 23.8 ± 1.3 (from 21 to 29) and in the group without dislocation 20.7 ± 1.2 (from 19 to 24), *p* = 0.008.

Preoperative diagnosis of zonule dehiscence was made in 13 patients with the dislocation group and in 7 patients without dislocation. Axial length of the eyeball in those patients was 23.8 ± 1.1 from 21 to 29 and 20.6 ± 0.8 from 19 to 22, respectively (*p* = 0.007).

Coexistent pathology is summarized in Table 2. There were no statistically significant differences in any of the pathologies like clinically visible pseudoexfoliation, degenerative myopia, uveitis, glaucoma or ocular hypertension, and diabetes mellitus.

Statistically significant difference was detected in the presence of the blunt eye trauma as a cause of lens subluxation. There were no cases in the dislocation group and 7 (26.9%) cases in the group without dislocation (*p* = 0.04).

Axial length of the eyeball was statistically significant higher in the group of patients with CTR/IOL complex dislocation. Two patients in this group had high degenerative myopia (axial length 28.4 mm and 29.5 mm, resp.) with myopic retinopathy.

In both cases high degenerative myopia may be regarded as the main factor responsible for zonule degeneration. It was possible that this two cases might have influenced the results. We performed second analysis of the group, having excluded this two cases of degenerative myopia and we repeated comparison with the control group. The results are summarized in Table 3. Statistical significance remained in respect of axial length of the eyeball (*p* = 0.009), IOL power (*p* = 0.002), refraction in spherical equivalent (*p* = 0.001), and preoperative diagnosis of zonule dehiscence (*p* = 0.007). There were no statistically significant differences in clinically visible pseudoexfoliation (*p* = 0.9).

## 4. Discussion

Our retrospective analysis indicates that higher axial length of the eyeball and associated myopia and low IOL power may be a strong predisposing factor to secondary dislocation of CTR/IOL complex.

We propose the hypothesis that longer axial length of the eyeball makes dislocation of the CTR/IOL complex more likely especially the presence of pseudoexfoliation and other previously identified factors like uveitis, glaucoma, and vitreo-retinal surgery. It is likely that what makes difference is the diameter of the CTR/IOL complex in relation to the diameter of the eyeball at the level of the ciliary processes. In cases where the diameter of the capsular bag stretched by the ring is significantly smaller than diameter of the eyeball at the level of ciliary body, where the CTR/IOL complex is fixated, CTR fails to release the tension of the ciliary processes adequately and does not prevent further degeneration of the ciliary processes and makes dislocation more likely.

There were no significant differences in the rate of pseudoexfoliation between the groups of patients with and without dislocation in our study.

Pseudoexfoliation is most frequent factor responsible for zonule weaknesses [14, 15], but our findings help to understand why some patients with lens subluxation secondary to pseudoexfoliation remain stable with CTR and some patients suffer from secondary dislocation of the CTR/IOL complex. It appears likely that axial length of the eyeball is one of the factors contributing.

Previously reported average time from primary surgery to dislocation was 6.8 years [2]. We aimed to look at the patients with rather early dislocation. We decided to analyze the patients who presented with dislocation within 5 years from primary surgery. This decision was based on the assumption that early secondary dislocation is the one that surgeon would most like to prevent, and these patients would be the ones more likely to reveal predisposing features. Also the 5-year period from primary surgery to dislocation was based on the practicability of obtaining the control group.

Composing a control group was a big challenge in this analysis, as it was difficult to obtain the patients who were operated on more than 5 years and still under the care of our department, to ascertain that no dislocation was present.

In our series none of the patients with CTR/IOL complex dislocation had blunt eye trauma as a reason for crystalline lens subluxation. Traumatic crystalline lens subluxations are due to mechanical rupture of the zonule in a specific area only and are not the reason for progressive zonule degeneration. These patients are good candidates for successful fixation with the CTR.

Zonule degeneration has many potential underlying genetic factors [16, 17]. Mutations in human fibrillin-1 and fibrillin-2, which are major constituents of tissue microfibrils, can affect multiple ocular components, including the ciliary zonule, lens, drainage apparatus, cornea, and retina.

In ectopia lentis observed in Marfan's syndrome [18], the fibers of the ciliary zonule appeared stretched or ruptured, consistent with a pathogenesis of haploinsufficiency, but in Weill-Marchesani syndrome, the problem appears to be aberrant formation of the zonule and the lens [18, 19]. Therefore, insights on the composition and formation of the zonule are especially valuable in the context of microfibril disorders. FBN1, FBN2, and FBN3 can form homo- or heterotypic microfibrils [20, 21].

Anatomical and mechanical factors in zonule degeneration are other important points for consideration [4].

The anatomy of the zonular apparatus and its structural arrangement have been described previously using ultrasound biomicroscopy [22, 23] and scanning electron microscopy [24].

The anterior zonule has a greater effect on the anterior lens surface, and the posterior zonule has a greater effect on the posterior lens surface. This may be due to the thickness difference between the anterior and posterior capsule [25] and, potentially, the role of the hyaloid membrane and its connections with the posterior zonule, which could act together to augment the effect of posterior zonular tension [26]. This may be another factor influencing the way CTR stretches the capsular bag following crystalline lens removal.

In myopia, the mechanism for lens subluxation may be different than in pseudoexfoliation [4, 27]. Lens subluxation may be the result of progressive overstretching of the processes, and progressive elongation of the fibers, rather than degeneration and weakening without elongation which is more likely in pseudoexfoliation. Elongation of zonular processes may result from the disproportion between the crystalline lens size and the size of the eyeball. Following phacoemulsification, and CTR insertion, the capsule becomes more flat, and its diameter becomes larger than before surgery. In myopic eye it may contribute to progress of the zonule degeneration.

Our study has several limitations. It was not possible to obtain data on the stage of crystalline lens subluxation preoperatively and intraoperatively. It appears likely that the more the degrees of subluxation, the more the chance of CTR failure to secure it. We cannot draw any conclusions regarding this aspect. Also the degree of zonule degeneration from pseudoexfoliation is not measurable at present and it is not possible to assess it unless subluxation is already present. CTRs were inserted by different surgeons, and there may be in-between surgeon variation in the approach to the subluxated crystalline lens, but this inconsistency may only have a minimal impact on the conclusions.

## 5. Conclusion

Axial length of the eyeball, myopia, and low IOL power appear to play a role in secondary CTR/IOL complex dislocation within 5 years from primary cataract surgery.

Patients with cataracts or lens subluxation as the result of blunt eye trauma appear likely to remain stable with CTR. Preoperative diagnosis of lens subluxation increases the chances of secondary IOL/CTR complex dislocation.

## Figures and Tables

**Table 1:** Comparison between patients of CTR/IOL complex dislocation within 5 years from lens surgery with control group of patients with no dislocation of CTR.

	Patients with CTR/IOL dislocation (*n* = 16 eyes)	Control group (*n* = 26 eyes)	(*p* value)
Age 28	66.2 ± 6.7 (from 49 to 82)	67.1 ± 7.2 (from 53 to 85)	0.7
M : F	10 : 5	17 : 5	0.8
Axial length of the eyeball (mm)	23.8 ± 1.3 (from 21 to 29)	20.7 ± 1.2 (from 19 to 24)	0.008
IOL power (dioptres)	10.2 ± 4.3 (from −3 to 19)	20.2 ± 1.6 (from 16 to 26)	0.0005
Refraction in spherical equivalent (*n* = 10)	−4.5 ± 2.8 (from −20.3 to −2.3)	+1.5 ± 1.6 (from −3.2 to +5.4)	0.0007
Preoperative diagnosis of zonule dehiscence	13 of 16 (81.5%)	7 of 26 (26.9%)	0.01
Clinically visible pseudoexfoliation	8 of 16 (50%)	15 of 26 (57.7%)	0.8

**Table 2:** Coexistent pathology in eyes of patients of CTR/IOL complex dislocation within 5 years from lens surgery with control group of patients with no dislocation of CTR.

	Patients with CTR/IOL dislocation (*n* = 16 eyes)	Control Group (*n* = 26 eyes)	*p* value
Clinically visible pseudoexfoliation (eyes)	8 (50%)	15 (57.7%)	0.8
Degenerative myopia (eyes)	2 (12.5%)	0 (0%)	0.6
Uveitis (patients)	2 (12.5%)	3 (11.5%)	0.8
Trauma before cataract surgery (eyes)	0 (0%)	7 (26.9%)	0.04
Glaucoma or ocular hypertension (eyes)	6 (37.5%)	12 (46.2%)	0.7
Diabetes (patients)	2 (12.5%)	7 (26.9%)	0.8

**Table 3:** Comparison between patients of CTR/IOL complex dislocation within 5 years from lens surgery, excluding 2 patients with degenerative myopia, with control group of patients with no dislocation of CTR.

	Patients with CTR/IOL dislocation (*n* = 14 eyes)	Control group (*n* = 26 eyes)	Chi-square (*p* value)
Age 28	67.3 ± 6.7 (from 49 to 82)	67.1 ± 7.2 (from 53 to 85)	0.7
M : F	18 : 5	17 : 5	0.8
Axial length of the eyeball (mm)	22.9 ± 1.2 (from 21 to 25)	20.7 ± 1.2 (from 19 to 24)	0.009
IOL power (dioptres)	10.2 ± 4.3 (from 14 to 19)	20.2 ± 1.6 (from 16 to 26)	0.002
Refraction in spherical equivalent (*n* = 9)	−4.2 ± 2.8 (from −6.3 to +2.3)	+1.5 ± 1.6 (from −3.2 to +5.4)	0.001
Preoperative diagnosis of zonule dehiscence (number of eyes)	11 of 14 (78.6%)	7 of 26 (26,9%)	0.007
Clinically visible pseudoexfoliation (number of eyes)	8 of 14 (57.1%)	15 of 26 (57.7%)	0.9
